# Pilot study on the therapeutic potential of radiofrequency magnetic fields: growth inhibition of implanted tumours in mice

**DOI:** 10.1038/s41416-020-0995-3

**Published:** 2020-07-20

**Authors:** Jukka Luukkonen, Jonne Naarala, Jukka Juutilainen, Frank Barnes, Carlos F. Martino

**Affiliations:** 1 Department of Environmental and Biological Sciences, University of Eastern Finland, Kuopio, Finland; 2 Department of Electrical, Computer, and Energy Engineering, University of Colorado, Boulder, CO USA; 3 Florida Institute of Technology, Biomedical and Chemical Engineering and Science, Melbourne, FL USA

**Keywords:** Cancer, Medical research

## Abstract

The present study investigated possible therapeutic effects of radiofrequency or hypomagnetic fields on the growth rate of two types of implanted tumours. To this end, mice with implanted fibrosarcoma and pancreatic tumours were exposed continuously to a 2 µT, 10 MHz radiofrequency magnetic field (MF) perpendicular to a 45 µT static MF or to a hypomagnetic (~0.4–1 µT) field. The reasoning for a 10 MHz treatment was based on a current theoretical explanation for MF effects, which predicts a resonance phenomenon in this frequency range. Radiofrequency MFs reduced consistently the growth rate of two implanted tumour types (by ~30% in both cases). Also, hypomagnetic field hindered tumour growth in both tumour types, but the observation was not statistically significant with fibrosarcoma tumours. In conclusion, although experiments included a limited number of animals, the results indicate that MFs may offer a novel therapeutic strategy in the treatment of cancer.

## BACKGROUND

Research on the biological effects of time-varying magnetic fields (MFs) has mostly focused on possible carcinogenicity and other adverse health effects of environmental 50–60 Hz MFs.^1^ However, it has also been proposed that time-varying MFs could be used as a therapeutic agent in the treatment of cancer, and some promising findings have been reported. Exposure to 100–120 Hz MFs—alone or in combination with ionising radiation—has been reported to retard the development of implanted mammary^2,3^ and liver tumours,^4^ as well as chemically induced preneoplastic liver lesions.^5^ However, the underlying mechanism for therapeutic effects of MFs is not known.

The radical pair mechanism (RPM) is considered to be a plausible biophysical mechanism for explaining the adverse health effects of weak environmental MFs,^6^ and this mechanism could also be involved in possible anti-carcinogenic effects of MFs. According to the RPM, chemical reactions with radical pairs as transient intermediates are sensitive to weak MFs, and therefore MFs could affect the concentration of intracellular free radicals.^7^ The RPM also predicts a resonance frequency range in the order of 0.1–10 MHz because hyperfine splittings occur in this range in most biomolecules.^7^ The effect should be larger when the static and oscillating field are perpendicular than when they are parallel to each other.

In this pilot study, we investigated potential tumour-suppressing effects of radiofrequency (RF) MFs. Mice bearing two types of implanted tumours were exposed to a 2 µT, 10 MHz MF perpendicular to a 45 µT static MF. Another treatment group was exposed to a hypomagnetic field (the ambient static geomagnetic field reduced to ~0.4–1 µT), based on earlier results suggesting that reduction of the geomagnetic field inhibits the growth of cultured cells.^8^

## METHODS

A total of 1 × 10^6^ cells/100 µL of either human fibrosarcoma HT1080 or pancreatic AsPC-1 cells were injected intradermally into the flank of 6-week-old female severe-combined immunodeficient mice (Taconic Farms Inc.) after anaesthesia by isoflurane inhalation at 2–3%. The growth of tumour volume was determined by the standard callipre-based measuring procedure twice per week. The measurement was done blinded, without knowledge of the exposure of the animals. In the beginning of the exposure (when the average volume of fibrosarcoma tumours exceeded ~50 mm^3^ and that of pancreatic cell tumours exceeded ~25 mm^3^), the mice were randomised by tumour volume and assigned to three treatment groups: (a) control (ambient geomagnetic field 45 µT), (b) hypomagnetic field (~0.4–1 µT) and (c) a 2 µT, 10 MHz RF MF in the horizontal direction combined with a vertical 45 µT static MF. The exposure system has been described in Supplementary Fig. 1. The hypomagnetic field was established by a horizontally resting open-ended µ-metal cylinder. The entire mouse cage was placed inside the µ-metal cylinder. The horizontal RF field and the vertical 45 µT static MF were established with square Helmholtz coils in tri-axial configuration, which were also used to cancel the horizontal component of the static geomagnetic field. Mice were continuously exposed to the treatments. Exposures were performed under the regulated environment set by the animal facility on all groups. Intensities of the static MFs were measured by a gaussmeter (Walker Scientific, Model FGM 4D2N, Joondalup, Australia). The RF MF was measured by a sensor comprised of two 1.5 cm turns in radius that was connected to an oscilloscope (Tektronix, TDS2014B, Beaverton, OR, USA). Tumour size, well-being, and body weight were observed twice per week. The experiments were terminated before tumour weight reached 10% of the animal weight. Mice were sacrificed by CO_2_ inhalation.

### Statistical analysis

A two- or three-way analysis of variance was used for statistical analysis, with MF treatment (RF or hypomagnetic) and a measurement time as fixed factors and cohort as a random factor (only in the case of fibrosarcoma tumours). Cohort was included as a random factor in the analysis because tumour growth rates differed statistically significantly between cohorts. Post-hoc tests were performed using Tukey’s honest significance test (SPSS for Windows version 25, IBM Corp., USA). A *p* value <0.05 was considered to be statistically significant.

## RESULTS

**Fig. 1:**
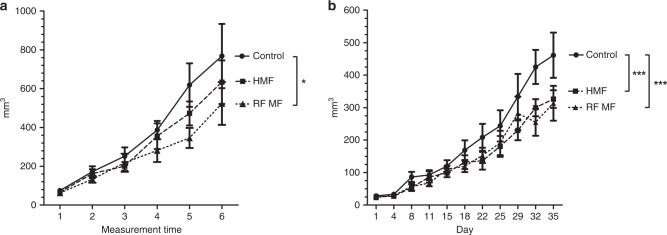
The effects of radiofrequency (RF) magnetic fields (MFs) and hypomagnetic field (HMF) on the volume of implanted tumors in mice. Mice were implanted with (**a**) fibrosarcoma and (**b**) pancreatic tumors and continuously exposed to horizontal 2 μT, 10 MHz RF MFs perpendicular to a 45 μT static MF, or HMF (~0.4–1 μT). Three independent experiments were performed using mice implanted with fibrosarcoma tumors. The total number of mice was 12 in the control group and 9 in each exposed group (4 + 3 + 3 in each experiment). Because of variations in initial tumor development, the tumor volume measurements did not occur exactly on the same days in the three fibrosarcoma tumor cohorts. Ordinal numbers from 1 to 6 therefore represent the measurement times (1: day 1; 2: days 4–6; 3: days 7–9; 4: days 11–13; 5: days 14–16; 6: days 18–20) in (**a**). In the pancreatic tumor experiment, four mice per group were exposed. *p < 0.05, ***p < 0.001.

The RF MF perpendicular to the static field inhibited the growth of implanted fibrosarcoma and pancreatic tumours in comparison to controls (Fig. 1). The effect size was similar in both xenograft models (an ~30% reduction). Also, hypomagnetic fields hindered the growth of implanted tumours, but the effect size was smaller than in the RF-exposed animals, and the effect on fibrosarcomas was not statistically significant. The MF treatments were highly tolerated; no significant whole animal weight loss or gain was observed compared to controls (data not shown), nor were there any observations suggesting reduction of well-being.

## DISCUSSION

In this study, we observed that a 2 µT, 10 MHz RF MF perpendicular to a 45 µT static field inhibited the growth of implanted fibrosarcoma and pancreatic tumours in mice. An ~30% reduction in tumour growth was consistently seen in both xenograft models, although the results of pancreatic tumour experiments must be interpreted with caution due to small group size. To the best of our knowledge, this is the first study suggesting that RF MFs in this frequency range affect tumour growth. The present results are concordant with previous results showing that RF MFs inhibit proliferation of HT1080 fibrosarcoma cells in vitro.^9^

The RF treatment resulted in consistent inhibition of tumour growth in two different xenograft models, but there is no clear understanding on the mechanism behind the observed effect. As the study was conducted using a frequency within the resonance range predicted by the RPM, the results can be considered to support the hypothesis that the underlying mechanism is MF effects on radical pairs. This hypothesis is supported also by the previous findings of increased radical level in HT1080 fibrosarcoma cells exposed to 10 MHz RF MFs.^9^ Positive findings in this frequency range fit both with the predictions of the RPM and experimental results in birds. Evidence for RPM-related biological effects of MFs is strongest in avian magnetoreception, and there is evidence that migratory birds can be prevented from using their magnetic compass when subjected to weak RF MFs with frequencies in the range 0.1–10 MHz.^7,10^ However, the current RPM theory is not able to account for the reported extraordinary sensitivity to RF fields.^7^

Also, the reduced growth of tumours in the hypomagnetic field, although not statistically significant in the fibrosarcoma model, might be explainable by the RPM: if the geomagnetic field is biologically effective in mammalian cells (like in RPM-related avian magnetoreception), removal of the geomagnetic field should result in biological effects. However, the avian magnetoreception system may have been optimised by evolution, and it is currently unclear whether the RPM explains sensitivity to weak MFs in mammalian cells.^1^

Although the limited number of animals used in the experiments restricts definite conclusions, the results of this pilot study indicate consistently that a 2 µT 10 MHz RF MF inhibits tumour growth in mouse models. Future studies are warranted to evaluate the applicability of RF MFs in tumour control and to optimise MF parameters (frequency, field strength and duration) for maximal therapeutic effects. Even the moderate effect size (~30%) observed in this study might be useful in combination with, for example, chemotherapy or radiotherapy.

## Supplementary information

Supplementary figure 1 legend

Supplementary figure 1

## Data Availability

The original raw data will be stored at UEF’s open access repository after publication.
