# Supplementary material for: Pilot study on the therapeutic potential of radiofrequency magnetic fields: growth inhibition of implanted tumours in mice
Source: Br J Cancer. 2020 Jul 20;123(7):1060–2. doi: 10.1038/s41416-020-0995-3 (PMC7524722; doi:10.1038/s41416-020-0995-3)
Supplement: Supplementary file 1 — Supplementary figure 1 legend [file 41416_2020_995_MOESM1_ESM.docx]

Supplementary Figure legend:

Supplementary Figure 1: A) Schematic of the exposure system. 1., 2., and 3. direct current (DC) power supplies (Power Designs, 1210S, Danbury, CT, USA; Hewlett Packard, 6024A, Palo Alto, CA, USA; Power Designs, 6050A, Danbury, CT, USA), 4. signal generator (SG) (Hewlett Packard, 33120A, Palo Alto, CA, USA), 5. oscilloscope (OSC) (Tektronix, TDS2014B, Beaverton, OR, USA), RF = radiofrequency. The oscilloscope was used to continuously monitor RF magnetic flux density during the exposures. The 1 ohm resistors are also shown in the diagram. B) 3-axial Helmholtz coil system (63.5 cm x 63.5 cm x 63.5 cm).
