# Supplementary figures and images for: Pilot study on the therapeutic potential of radiofrequency magnetic fields: growth inhibition of implanted tumours in mice
Source: Br J Cancer. 2020 Jul 20;123(7):1060–2. doi: 10.1038/s41416-020-0995-3 (PMC7524722; doi:10.1038/s41416-020-0995-3)

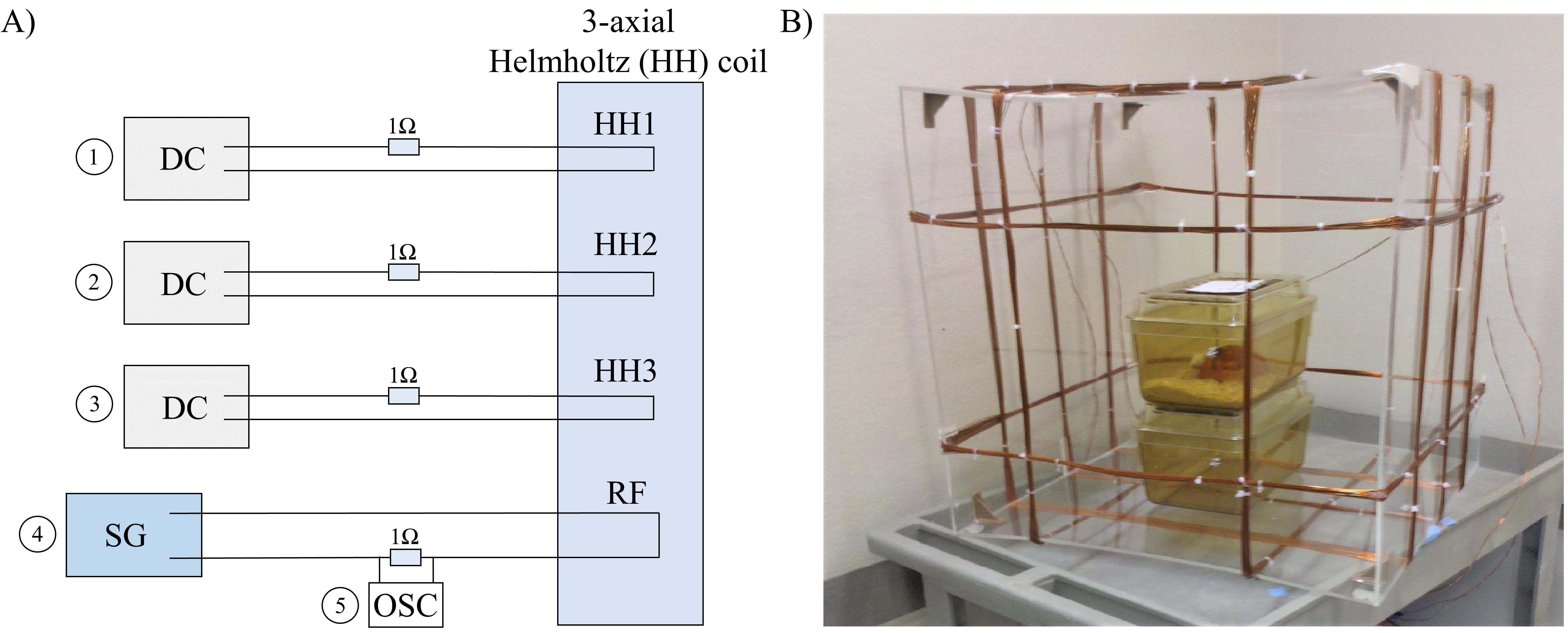

Supplement: Supplementary file 2 — Supplementary figure 1 [file 41416_2020_995_MOESM2_ESM.tif]
